# Cutaneous and mucosal human papillomaviruses differ in net surface charge, potential impact on tropism

**DOI:** 10.1186/1743-422X-5-118

**Published:** 2008-10-14

**Authors:** Nitesh Mistry, Carl Wibom, Magnus Evander

**Affiliations:** 1 Department of Virology, Umeå University, SE-901 85, Umeå, Sweden

## Abstract

Papillomaviruses can roughly be divided into two tropism groups, those infecting the skin, including the genus beta PVs, and those infecting the mucosa, predominantly genus alpha PVs. The L1 capsid protein determines the phylogenetic separation between beta types and alpha types and the L1 protein is most probably responsible for the first interaction with the cell surface. Virus entry is a known determinant for tissue tropism and to study if interactions of the viral capsid with the cell surface could affect HPV tropism, the net surface charge of the HPV L1 capsid proteins was analyzed and HPV-16 (alpha) and HPV-5 (beta) with a mucosal and cutaneous tropism respectively were used to study heparin inhibition of uptake. The negatively charged L1 proteins were all found among HPVs with cutaneous tropism from the beta- and gamma-PV genus, while all alpha HPVs were positively charged at pH 7.4. The linear sequence of the HPV-5 L1 capsid protein had a predicted isoelectric point (pI) of 6.59 and a charge of -2.74 at pH 7.4, while HPV-16 had a pI of 7.95 with a charge of +2.98, suggesting no interaction between HPV-5 and the highly negative charged heparin. Furthermore, 3D-modelling indicated that HPV-5 L1 exposed more negatively charged amino acids than HPV-16. Uptake of HPV-5 (beta) and HPV-16 (alpha) was studied *in vitro *by using a pseudovirus (PsV) assay. Uptake of HPV-5 PsV was not inhibited by heparin in C33A cells and only minor inhibition was detected in HaCaT cells. HPV-16 PsV uptake was significantly more inhibited by heparin in both cells and completely blocked in C33A cells.

## Findings

Papillomavirus (PV) belongs to the *Papillomaviridae *family and consists of a large family of non-enveloped double stranded DNA viruses that infect the basal layer of cutaneous or mucosal epithelia of a dozen vertebrate species with a strict species tropism [1]. Over 100 human papillomavirus (HPV) types have been completely described and identified in human tissues and they together with animal PVs are divided into 16 genera based on their nucleotide sequence identity of the major capsid protein L1 open reading frame (ORF) [2]. HPVs can roughly be divided into two tropism groups, those infecting the skin, including the genus beta PVs, and those infecting the mucosa, predominantly genus alpha PVs.

The first step of PV infection is binding of the major capsid protein L1 to the cell surface. The cell surface glycosaminoglycan (GAG) heparan sulfate (HS) is important for HPV infection *in vitro *of several HPV types from the alpha genus [3-7]. HS contains highly sulphated repeats of disaccharides and is highly negatively charged. Other receptors than HS may function at a later entry step and α6-integrin has been suggested as a candidate receptor [8]. Furthermore, PVs has been shown to bind to a basal extracellular matrix component that co-localizes with laminin-5 within the basal extracellular matrix [9-11]. Since the sequence of the L1 capsid protein determines the phylogenetic separation between beta types and alpha types [2] and the L1 protein is most probably responsible for the first interaction with the cell surface, one can suspect that the interactions of the viral capsid with the cell surface could affect HPV tropism. To study this, the net surface charge of the HPV L1 capsid proteins was analyzed and HPV-16 (alpha) and HPV-5 (beta) with a mucosal and cutaneous tropism respectively, were used to study heparin inhibition of uptake.

The predicted isoelectric point (pI) of the receptor binding L1 capsid for all available HPV types was examined and found to correlate with the cutaneous or mucosal tropism (Table 1). The pI calculations were based solely on protein sequence and the charge of exposed epitopes of L1 were not calculated. Interestingly, the negatively charged L1 proteins were all found among HPVs with cutaneous tropism from the beta- and gamma-PV genus (Table 1). The pI was almost identical for HPV types within the same species. HPV-5 had a pI of 6.59 and a negative charge of -2.74 at pH 7.4. In comparison the alpha HPV-16, which interacts with the negatively charged heparan sulfate, had a pI of 7.95 and a +2.98 positive charge at pH 7.4. In fact all alpha HPVs from the alpha-PV genus were positively charged at pH 7.4 (Table 1). The exception was HPV-2 and HPV-27 from the alpha-PV genus species 4, but they have been detected both in cutaneous and mucosal lesions [2].

**Table 1:** Predicted values for pI and charge at pH 7.4 for HPV L1.

Genus	Species	Infected region	HPV type	pI-value	Charge at pH 7.4
Alpha-papillomavirus	1	Mucosa	32	8.38	5.2
	
	2	Cutaneous (Mucosa)	10	7.65	1.02
	
	3	Mucosa	61	8.2	4.88
	
	4	Cutaneous (Mucosa)	2	6.87	-1.8
	
	5	Mucosa	26	8.38	5.24
	
	6	Mucosa	53	8.59	6.3
	
	7	Mucosa	18	8.35	7.85
	
	8	Mucosa (Cutaneous)	7	7.46	0.2
	
	9	Mucosa	16	7.95	2.98
	
	10	Mucosa	6	8.55	7.08
	
	11	Mucosa	34	8.3	6.68
	
	13	Mucosa	54	8.5	6.15

Beta-papillomavirus	1	Cutaneous	5	6.59	-2.74
	
	2	Cutaneous	9	5.88	-6.76
	
	3	Cutaneous	49	6.01	-4.94

Gamma-papillomavirus	1	Cutaneous	4	6.34	-4.43
	
	2	Cutaneous	48	5.57	-7.53
	
	3	Cutaneous	50	6.02	-5.57
	
	4	Cutaneous	60	7.14	-0.71

Mu-papillomavirus	1	Cutaneous	1	6.63	-1.54

	2	Cutaneous	63	6.56	-2.43

Nu-papillomavirus	1	Cutaneous	41	6.13	-6.71

The HPV types displayed are the HPV type species according to[2]. The other HPVs in the species had similar pI as the type species (data not shown). The theoretical isoelectric point (pI) calculations in this study were based solely on protein sequence and did not take 3D-aspects or residue-residue interactions into consideration. To attain a prediction of the pI of the protein the theoretical charge was calculated at every pH between 1 and 13, with an increment of 0.01. The pH with the total charge closest to zero was used as the pI of the protein. The theoretical charge of a sequence at any given pH was determined by the frequency of a few amino acids. Lysine, arginine, histidine and the N-terminal residue contributed positively to the over all charge of a sequence, whereas aspartic acid, glutamic acid, cysteine, tyrosine as well as the C-terminal residue contributed negatively. All other types of amino acids were for this purpose considered neutral. To attain the overall charge of the protein, the total contributing charge for each of the positive amino acids was summarized and the total contributing charge for all negative amino acids was subtracted . The pKa-constants we employed for our calculations were kindly shared to us by the European Molecular Biology Laboratory.

The interaction of HPV with HS seems to depend on charge distribution since replacement of three surface-exposed lysine residues for alanine in the HPV-16 L1 capsid protein resulted in reduced cell binding and infectivity of HPV-16 PsV [12]. The authors suggested that these lysine residues cooperate in a charge-dependent HS binding, facilitation HPV-16 PsV infection [12]. These residues are conserved among alpha-PVs, but are not found in the beta HPVs.

**Figure 1:**
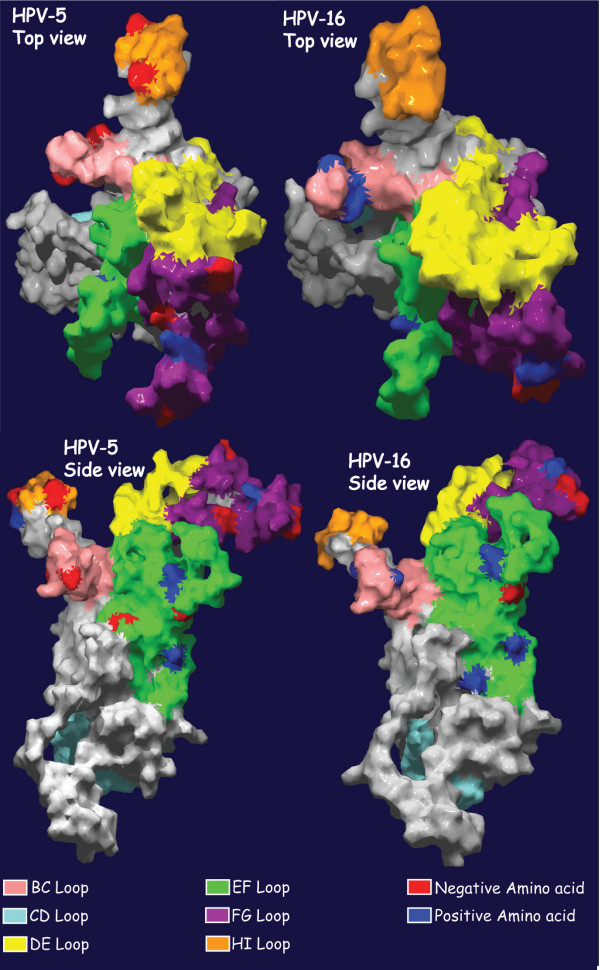
**Comparison of surface exposed charge differences of HPV-5 and HPV-16 L1 capsid proteins**. The model of the HPV-5 L1 monomer is overlaid on the crystal structure of HPV-16 L1. Colour in this illustration display positive (blue) and negative (red) amino acids loops of L1. To model L1 capsid protein the structure file for the HPV-16 L1 monomer, 1 DZL.pdb [13], was downloaded and used as a template to model L1-monomers for HPV-5 using SWISS MODEL . Protein models were also ray-traced using POV-Ray . The 3D-models of L1 protein of an alpha-papillomavirus (HPV-16) and a beta-papillomavirus (HPV-5) visualize the number of charged amino acids on one L1 capsid protein that are more than 30% exposed in the surface when grouped into a pentamer.

A 3D-model of the L1 protein monomer of the alpha HPV-16 and the beta HPV-5 was generated by using the structure file for the HPV-16 L1 monomer [13] to visualize the number of exposed charged amino acids on L1 protein. The 3D-model showed that each HPV-5 L1 monomer contained 4 amino acids with a positive charge and 9 with a negative charge on the different loops. HPV-16 L1 had 6 positive and only 3 negative charged amino acids exposed on its surface (Figure 1). The invading arms were also studied and showed only a small difference in exposed charged amino acids. HPV-5 had 10 positive and 12 negative amino acids whereas HPV-16 had 11 positive and 8 negatively charged amino acids on the part of the invading arm exposed to the surface (data not shown). Given that there are 360 L1 monomers to build up a HPV capsid these result implied that HPV-5 was more negatively charged than HPV-16. The HPV-5 L1 monomer was generated using the HPV-16 L1 monomer as a template, and therefore these calculations needs to be further addressed when more PV capsids have been crystallized to fully evaluate the epitopes.

The difference in theoretical net surface charge between the L1 protein of alpha and beta PVs prompted an analysis of dependence of charged receptor structures for virus uptake into cells.

Heparin is highly negatively charged and was used for inhibition studies of alpha HPV-16 and beta HPV-5 pseudovirus (PsV) uptake, analyzed by expression of the GFP marker gene [14,15]. GFP-expressing PsV were produced according to established methods [16]. Briefly, plasmids expressing the codon-modified papillomavirus major and minor capsid proteins, L1 and L2, together with a green fluorescent protein (GFP) expressing reporter plasmid, were transfected into 293TT cells. Capsids were allowed to mature overnight in cell lysate and were then purified using OptiPrep^® ^gradients (Axis-Shield). Plasmids and 293TT cells used for pseudovirus production were a kind gift from Chris Buck (NCI, Bethesda, Maryland, USA) and Martin Müller (German Cancer Research Center, Heidelberg, Germany). Detailed protocols are available at the website . The human epithelial cell line HaCaT, from adult trunk skin [17] and the human cervical cell line C33A [18] were plated at 5 × 10^4 ^cells/well in 24-well plates. PsV doses were calibrated according to [19] and before addition to the cells, PsV were incubated with various concentrations of heparin (Sigma Chemical Co.) for 60 min on ice in DMEM with 10% FCS. The PsV and heparin solution were then added to cells for 44–52 h at 37°C and fluorescence was quantified by flow cytometry [19]. There was no significant heparin inhibition of HPV-5 PsV uptake in cervical C33A cells and only a small inhibitory effect of heparin on HPV-5 PsV mediated GFP expression was noted in cutaneous HaCaT cells. Uptake of HPV-16 PsV mediated GFP expression was completely inhibited in C33A cells at 30 μg/ml of heparin (IC_50 _= 15.5 μg/ml) and it was also dose-dependent (IC_50 _= 24.5 μg/ml) with a maximum inhibition level of 72% in HaCaT cells. The difference in inhibition between HPV-5 and HPV-16 was significant (using a 2-tailed student *T*-test) both in C33A cells (P < 0.0004) and in HaCaT cells (P < 0.03).

In summary, beta HPV-5 PsV uptake was not inhibited in C33A cells and there was only a minor inhibition detected in HaCaT cells similar to previous results where HPV-5 pseudovirus infection of HeLa cells was not inhibited by heparin [20]. Alpha HPV-16 was significantly more inhibited by heparin in both cells and completely blocked in C33A cells, similar to another alpha-PV, HPV-31b, where infection in C33A cells was clearly dependent on heparan sulfate, while pre-treatment with heparin only had a small inhibitory effect of infection in HaCaT cells [21].

In a study comparing HS expression in skin and mucosa, more GAGs were found in vaginal tissue than in perianal skin, although this difference could possibly be explained by differential expression of chondroitin sulfates and dermatan sulfate and not HS [22]. Both the extracellular matrix in addition to the cell surface and the epithelial basement membrane seems to be the primary site of virus binding during genital tract infection *in vivo *[23] and a dynamic model for alpha-HPV entry has been suggested where binding of alpha HPV to cell surface HS results in conformational change in the capsid, followed by binding to a second receptor [24]. This second receptor is suggested to be a non-HS receptor [25] and is most probably L1-specific [24]. Tropism could also be determined at another step in the infectious cycle and the long control region (LCR) of HPV DNA, which contains binding sites for transcription factors and numerous binding sites for epithelial specific enhancers has been studied for a role in HPV tropism[26]. In HaCaT cells, the HPV-5 LCR was two-fold more efficient in transcriptional activation compared to the HPV-16 LCR, while in cervical W12E cells the HPV-16 LCR was almost 2-fold more effective in activating transcription compared to the HPV-5 LCR [27].

To conclude, the first step of a virus infection is attachment to the cell surface and net charge is important for determining virus binding to cell receptors, as has been shown for certain adenoviruses [28]. The observed differences between HPV-5 and HPV-16 could be of importance for PV tropism, but extended studies with more HPV types, including the possible second receptors have to be performed.

## Competing interests

The authors declare that they have no competing interests.

## Authors' contributions

NM participated in the design of the study, carried out virus infection experiments, 3D modelling and drafted the manuscript. CW carried out calculation of theoretical pI and 3D modelling experiments. ME conceived the study, participated in its design and coordination and helped to draft the manuscript. All authors read and approved the final manuscript.
